# The Effects of Milk Replacer Supplemented with *Ascophyllum nodosum* as a Novel Ingredient to Prevent Neonatal Diarrhea in Dairy Calves and Improve Their Health Status

**DOI:** 10.3390/vetsci10100618

**Published:** 2023-10-13

**Authors:** Elena Scaglia, Serena Reggi, Benedetta Canala, Sara Frazzini, Matteo Dell’Anno, Monika Hejna, Luciana Rossi

**Affiliations:** 1 Department Civil, Environmental, Architectural Engineering and Mathematics—DICATAM, University of Brescia, 25123 Brescia, Italy; elena.scaglia@unibs.it; 2 Department of Veterinary Medicine and Animal Sciences—DIVAS, University of Milan, 26900 Lodi, Italy; serena.reggi@unimi.it (S.R.); benedetta.canala@unimi.it (B.C.); sara.frazzini@unimi.it (S.F.); matteo.dellanno@unimi.it (M.D.); 3 Department of Biotechnology and Nutrigenomics, Institute of Genetics and Animal Biotechnology of the Polish Academy of Sciences, Jastrzębiec, 05-552 Magdalenka, Poland; m.hejna@igbzpan.pl

**Keywords:** *Ascophyllum nodosum*, calves, neonatal diarrhea, novel ingredients, gut health, alternative to antibiotics

## Abstract

**Simple Summary:**

The pre-weaning period in calves is a critical phase in cattle farming, where neonatal calf diarrhea can affect animal welfare, leading to death in the most severe cases. Improving animal health is necessary to reduce antibiotic use, thereby reducing antibiotic resistance. Functional ingredients such as brown seaweeds could be integrated into the calf’s nutritional plan for preventive purposes to increase gut health and metabolism. These seaweeds have a high content of bioactive compounds, such as polysaccharides, polyunsaturated fatty acids, antioxidants, peptides, vitamins, and minerals. This study evaluated the effects of using a macroalgae, *Ascophyllum nodosum*, as a supplement in pre-weaning calf nutrition on zootechnical performance, blood metabolism, and fecal bacteria. It was found to be particularly effective in cases of moderate diarrhea.

**Abstract:**

Nutrition and health during pre-weaning affect the calves’ future fertility, calving age, production, and carrier length. Calves are highly susceptible to neonatal calf diarrhea (NCD), which can be fatal. NCD is due to hypovolemia and acidosis, which may involve anorexia and ataxia. The One Health principle calls for a drastic reduction in antimicrobial use. One approach is to improve animal health and reduce the use of antibiotics and functional ingredients that have beneficial effects due to bioactive compounds. Several functional ingredients and additives can be considered, and, in particular for this study, *Ascophyllum nodosum* was considered. The present study aimed to evaluate the role of *A. nodosum* as a functional ingredient implemented into the milk replacer in neonatal calves. Twelve pre-weaned Holstein Frisian calves, housed in twelve individual pens in the same environmental conditions, were divided into two groups of six animals: a control group (CTRL, *n* = 6) fed with a milk replacer, and a treatment group receiving milk enriched with 10 g of *A. nodosum* in their diet (TRT, *n* = 6) for 42 days. The fecal score was evaluated daily (3–0 scale) to monitor the incidence of diarrhea in the two groups. The body weight was evaluated weekly, and every two weeks feces were collected for microbiological evaluation using a selective medium for plate counting of total, lactic acid, and coliform bacteria. To verify the presence of *Lactobacillus*, *Bifidobacterium*, and *Escherichia coli*, real-time qPCR was used. At the beginning and at the end of the trial, blood samples were obtained for serum metabolite analysis. The growth performance did not differ in either of the two groups, but significant differences were observed in the incidence of moderate diarrhea (*p-*value < 0.0113), where the TRT group showed a lower incidence of cases during the 42-day period. Serum analysis highlighted higher contents of albumin, calcium, phosphorus, and total cholesterol in the TRT group compared to CTRL (*p-*value < 0.05). In conclusion, implementation of *A. nodosum* in the diet of calves can lead to better animal welfare and may reduce the use of antibiotics.

## 1. Introduction

The health status of calves has maximum attention during the pre-weaning period, with positive effects for the herd’s economy, ensuring the well-being and regular growth performance of calves and better milk quality and production in terms of future perspectives. Managerial success in this phase of life allows the overcoming of future and current challenges. Colostrum is the first strategy that allows calves to obtain passive immunity, ensuring animal health [1]. Despite progress in understanding the physiopathology of intestinal disease, neonatal diarrhea remains the principal cause of illness in dairy calves. The first period in calves’ lives is crucial due to increased susceptibility to pathogens [2]. Numerous infectious agents are involved in calf diarrhea, and the group of enteric pathogens is the most representative. *Escherichia coli*, one of the most common, is characterized by several pathotypes and represents a health challenge for calves raised worldwide [3]. The incidence of neonatal diarrhea in calves is due to a series of complex factors, including infectious agents and environmental aspects, and animal welfare should also be considered. If the calves are in poor health, this leads to lower growth performance, higher antimicrobial use, and significant costs for animal care [4,5]. Furthermore, if diarrhea is not appropriately managed, there may be increased mortality rates due to hypovolemia and acidosis, leading to anorexia and ataxia in the calves [6]. Due to high antibiotic resistance, drugs should be used following the 3Rs principle approved by the European Food Safety Authority (EFSA), and antibiotic alternatives are required [7,8]. Functional ingredients and additives can reduce the spread of antimicrobial resistance and help prevent long-lasting immunocompromising effects on the intestinal microbiome with consequent digestive tract dysfunctions. During pre-weaning, farm management plays a role in: (i) preventing diseases from spreading; (ii) ensuring future profitability, productivity, and the fertility of the herd; and (iii) reducing antimicrobial use [9,10]. The European Food Safety Authority (EFSA) has highlighted the importance of promoting new strategies that could reduce the effects of diarrhea and protect global health from a One Health perspective [11]. One approach to increase animal health and reduce the use of antibiotics is to exploit natural organisms with functional properties due to containing bioactive compounds [6,12]. One such natural organism is seaweed, also known as macroalgae, a heterogeneous group of pluricellular marine organisms rich in bioactive substances such as polysaccharides, proteins, lipids, and polyphenols that give them antibacterial, antiviral, and antifungal properties [13,14]. Many studies on animal nutrition have reported the antioxidant effects of macroalgae due to the high content of polyphenols, bioactive compounds, minerals (I, K, Ca, Mg, P, Fe, and Zn), and vitamins (C, B1, B2, and E), which are higher than in microalgae and cyanobacteria. Macroalgae also have antimicrobial effects, especially towards *E. coli* [13]. However, the heterogeneity of the macroalgae group influences the inclusion levels in the diet due to the different properties and biochemical composition [15,16]. There appear to be no adverse effects on monogastric animals’ performance and physiological parameters. There have been positive outcomes in treating pigs during weaning [17], and including such additives in pig feed positively impacts gut health [18]. Although monogastric species have been investigated in some depth, this is different for ruminant species. Our aim was thus to evaluate *Ascophyllum nodosum* and its effects as an additive in milk replacer to dairy calves during pre-weaning to test animal health, growth performance, incidence of diarrhea, and blood parameters.

## 2. Materials and Methods

### 2.1. Animals, Housing, Experimental Design and Treatment

The experimental trial was approved by the Animal Welfare Organization of the University of Milan (OPBA authorization 129/2021) and performed following European regulations in an intensive dairy farm in the north of Italy.

A total of 12 pre-weaned Holstein Frisian calves were involved in the trial at the same period of life (under one week of age), housed in individual pens, and maintained under homogeneous environmental conditions for the entire duration of the experiment. The dimensions of each pen complied with EU regulations [19]. The calves were administered a standard quantity (4 L) of colostrum within four hours after birth, and the subsequent study lasted 42 days. The quality of colostrum was assessed at 27% using a Brix refractometer. The calves were divided into two balanced groups considering the initial weight (37.79 ± 4.900 kg), the age (all the subjects had an age under one week of life when involved in the trial), and the sex (50% male, 50% female) to re-create the conditions typically present in livestock, where the pathologies related to the pre-weaning period affect both males and females. The experimental group received the same basal diet; specifically, the control group (CTRL, *n* = 6) was fed with milk replacer (Gruppo Veronesi S.p.a., Verona, Italy) (Table 1), and the treatment group (TRT, *n* = 6) was fed with milk replacer supplemented with 10 g/day of *A. nodosum* powder (Italfeed S.r.l., Milan, Italy). The inclusion rate was established, based on previous studies [20] that included A. nodosum and brown algae at 0.2–0.3% in monogastric feed [21,22], aiming to increase the palatability and ensuring a proper milk suspension due to the partial solubility of algal powder. The *A. nodosum* used in the present study contained 92% of dry matter (DM), 21.41% of ash, 8.25% of crude protein (CP), 3.3% of ether extract (EE), and 3.57% of crude fiber (CF). The nutrient composition of the alga was analyzed following the official methods (AOAC, 2019) for the evaluation of DM, CP, EE, and ash content. Each parameter has been determined in triplicate. The DM was determined after drying the alga in a forced air oven at 65 °C for 24 h (AOAC method 930.15). Nitrogen content was determined using the Kjeldahl method (AOAC method 2001.11), and CP content was calculated as N × 6.25. The EE was obtained with a Soxhlet system, using an ether extraction (AOAC method 2003.05). Finally, ash content was obtained after incineration in a muffle furnace at 550 °C (AOAC method 942.05). 

The animals were fed twice a day. At the beginning of the trial, calves received 4 L of milk replacer equally distributed for the two meals (quantity of powder = 100g/L as indicated by the manufacturers Lattover, Veronesi Verona S.p.A., Verona, Italy), which was increased every week following the nutritional guidelines for calves [19,23] and their growth curve [24]. Fecal score was checked daily for the presence of diarrhea. The fecal score was assessed using a four-level scale: 0 = normal consistency (feces firm and well-formed); 1 = soft consistency (feces soft and formed); 2 = mild diarrhea (fluid and yellowish); 3 = severe diarrhea (fluid and projectile) [25]. A fecal score ≤ 1 was considered normal, whereas a fecal > 1 was defined as diarrhea. Moreover, in order to monitor the animal welfare, the health status was also screened daily by observing the vitality of the animals [26]. Each animal was individually weighed once a week, and the feed intake was evaluated by measuring the feed refused. Average daily feed intake (ADFI) was calculated. Every two weeks, feces were collected in sterile tubes and stored at −20 °C until the DNA extraction. The sampling was performed after the administration of the morning meal through rectal stimulation. Blood samples were collected on days 0 and 42, before the administration of the morning meal, from the jugular vein using 10 mL vacuum tubes without any anticoagulant. After the sampling, the blood was allowed to clot at room temperature and centrifugated at 3000 rpm for 15 min at 4 °C to obtain the serum that was immediately stored at −20 °C.

### 2.2. Metabolic Profile, Antioxidant Barrier, and Immunoenzymatic Analysis in Serum Samples

#### 2.2.1. Metabolic Profile

The serum aliquots were analyzed using a multiparametric autoanalyzer for clinical chemistry (ILab 650; Instrumentation Laboratory Company, Lexington, MA, USA), and the following parameters were considered: albumin (g/L); globulin (g/L); albumin/globulin (A/G ratio); beta-hydroxybutyrate (mmol/L); gamma-glutamyl transferase (IU/L); non-esterified fatty acids (mmol/L); glucose (mmol/L); urea (mmol/L); total bilirubin (µmol/L); total cholesterol (mmol/L); triglycerides (mmol/L); phosphorus (mmol/L); calcium (mmol/L); and magnesium (mmol/L). The serums were analyzed by the Experimental Institute of Lombardy and Emilia Romagna (IZSLER).

#### 2.2.2. Antioxidant Barrier

The oxidative status of the calf serum was evaluated through two commercial kits: an OXY-Adsorbent test and a dROMs test (DIACRON INTERNATIONAL research and diagnostic, Grosseto, Italy). The OXY-Adsorbent test was used to evaluate the capacity of a serum sample to counteract a massive oxidant insult in vitro inducted from a solution of HClO. A chromogenic technique was used to obtain a numerical result through a photometric reading. The dROMs test measures the oxidant capacity towards a modified aromatic amine used for its chromogen effect (DIACRON, 2022). The absorbance of each sample was read at a wavelength of 546 nm through a spectrophotometer (V630 UV–Vis, Jasco GmBH, Pfungstadt, Germany).

#### 2.2.3. Immunoenzymatic Tests for Serum Concentration of Trefoil Factor 3 and Diamine Oxidase

Immunoenzymatic tests and the enzyme-linked immunoassay (ELISA) were used to analyze the serum and thus highlight the animals’ health status by indirect markers. Specific kits were used for the detection of diamine oxidase (DAO) and trefoil factor 3 (TFF-3) as indirect markers of gut integrity directly implicated in the intestinal integrity of calves (Li StarFish S.r.l., Milan, Italy).

### 2.3. Microbiological and Molecular Analysis of Fecal Samples

#### 2.3.1. Bacterial Count

After collection, the feces were placed at 4 °C overnight, and plate counting was performed. One gram of each fecal sample was homogenized with 9 mL of sterile physiological solution and centrifuged (3000 rpm for 10 min at RT) to collect the supernatant. Samples were progressively diluted up to 10^−10^ [27] and then plated in three replicates in Petri dishes to count the total bacteria, lactic acid bacteria, and coliform bacteria using:

(i) Total bacteria: Plate Count Agar (PCA) (Liofilchem, Teramo, Italy). Incubation lasted three days at 30 °C (Merck, Taufkirchen, Germany);
(ii) Lactic acid bacteria: de Man, Rogosa and Sharpe Agar (MRSA) (Liofilchem, Teramo, Italy). Incubation lasted three days at 30 °C under microaerophilic conditions (Merck, Taufkirchen, Germany);
(iii) Coliform bacteria: Violet Red Bile Lactose Agar (VRBLA) (Liofilchem, Teramo, Italy). Incubation lasted 18–24 h at 35 °C under microaerophilic conditions (Merck, Taufkirchen, Germany).

All the results were expressed as log_10_ of colony-forming units per gram of fresh feces (Log_10_ CFU/g).

#### 2.3.2. Bacterial DNA Extraction and Real-Time PCR

Fecal swabs were collected every two weeks and stored at −20 °C until further processing. The total DNA was extracted from swabs using the QIAamp Power Faecal Pro DNA kit (QIAGEN, Düsseldorf, Germany) using the manufacturer’s instructions. DNA concentration and DNA quality were evaluated using a Nanodrop BioteK Synergy HTX spectrophotometer (Agilent, Carpinteria, CA, USA). A real-time PCR was conducted to evaluate the relative abundances of different bacterial populations. *E. coli* was used as representative pathogenic bacteria and *Lactobacillus* spp. and *Bifbifidobacterium* spp. as beneficial bacteria (Table 2). A comparative ∆∆CT approach was used in the real-time PCR, while the quantification of total bacteria was used as an endogenous control [28]. The qRT-PCR was carried out with the CFX Opus 96 (BioRAD, Richmond, CA, USA). The PCR mixing was performed in a total of 25 μL containing 12.5 μL of 2X SsoAdvanced Universal SYBR Green Supermix (BioRad, Richmond, CA, USA), 0.5 µM of each primer, and 50 ng of DNA template. The parameters for the amplification were as follows: for the *Lactobacillus* spp. used for an initial denaturation of 2′ at 98 °C, followed by 40 cycles of 15″ at 98 °C, 30″ of annealing at 56.5 °C and 40″ of extension at 60 °C. Instead, the amplification of total bacteria, *E. coli* spp. and *Bifidobacterium* spp. were the same for an initial denaturation of 2′ at 98 °C, followed by 40 cycles of 15″ at 9 °C and, 40″ of annealing/extension at 60 °C. The melting curve for all the amplification was determined in 60–95 °C range with increments every 5′ of 0.5 °C.

### 2.4. Statistical Analysis

The number of animals (six for each group) was defined using the GPower software, version 3.1.9.7., considering an observable difference of two independent samples for a power test set at 80%, a protection level of 95%, and an effect size of 1.12%.

Data analysis was conducted using GraphPad Prism (v. 9.00, Boston, MA, USA). The normality distribution of the data was evaluated by the Shapiro–Wilk test. Zootechnical performance, fecal score data, and fecal bacterial counts were analyzed using a repeated-measures two-way ANOVA. The results were evaluated using a full factorial model (Treatment: Trt, Time: Time, Interaction: Trt × Time). Daily data of feed intake were analyzed using the weekly average for each calf. Multiple comparisons among groups were evaluated by performing Tukey’s honest significance difference test (Tukey’s HSD).

To adjust the initial variability of serum samples, serum metabolite data, OXY and dROMs tests, and ELISA results were evaluated after analyzing the covariances (ANCOVA) to adjust for the initial individual variability. The results were statistically different at *p-*values lower than 0.05 and are presented as the mean ± standard error or standard deviation.

## 3. Results

### 3.1. Zootechnical Performances

**Figure 1:**
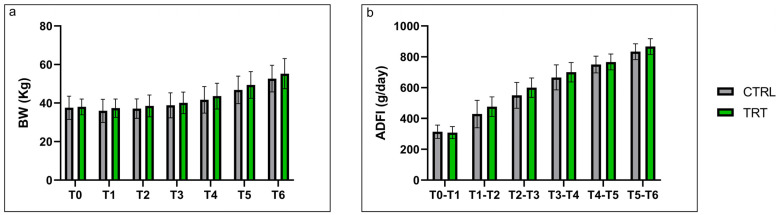
Zootechnical performance of control (CTRL) and treatment groups (TRT) measured over 42 days of experimental trial including the animal’s body weight over time (**a**), and the average daily feed intake averaged for each week of trial over time (**b**). Data are expressed as means ± standard deviation (SD). Abbreviations: BW = body weight; ADFI = average daily feed intake.

The seaweed supplementation in the milk replacer did not influence the performance of pre-weaning calves (Figure 1). The treatment did not affect the acceptability of the animals’ diet; the experimental groups showed similar average daily feed intakes (ADFI) (Figure 1b). The results showed a constant and comparable increase in body weight in both experimental groups during the entire trial period (42 days). As for the other zootechnical parameters, ADG did not differ between TRT and CTRL groups and constantly increased from 0.16 ± 0.235 kg at T1, reaching 0.84 ± 0.200 kg at T6 for the CTRL group and from 0.17 ± 0.191 kg to 0.84 ± 0.260 kg for the TRT group. Also, FCR did not differ between TRT and CTRL groups, starting from 5.34 ± 14.870 kg at T1 and reaching 1.05 ± 0.286 kg at T6 for the CTRL group and starting from 7.45 ± 7.480 kg and reaching 1.12 ± 0.349 kg for the TRT group.

### 3.2. Metabolic Profile, Antioxidant Barrier, and Immunoenzymatic Analysis of Serum Samples

#### 3.2.1. Metabolic Profile

Analysis of the metabolic profile in the TRT group showed that the albumin, calcium, phosphorus, and total cholesterol levels (*p* < 0.05; Table 3) were significantly higher than in the CTRL group.

#### 3.2.2. Oxidative Status of Blood Serum

**Figure 2:**
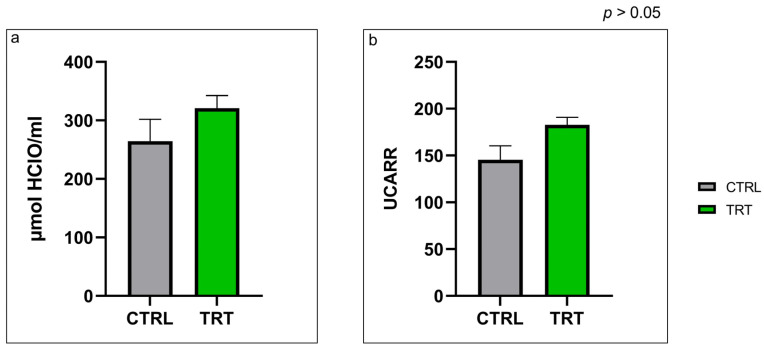
Oxidant serum status for control (CTRL) and treatment (TRT) groups at day 42 of the trial, including OXY-adsorbent test on serum samples (µmol HClO/mL) (**a**) and d-ROMs test (UCARR) (**b**). Data are expressed as means ± standard deviation (SD).

Data from the OXY-adsorbent test and the d-ROMs test revealed no significant differences between experimental groups after 42 days of the trial (Figure 2). The OXY-adsorbent test value at the end of the trial was 264.40 ± 91.781 µmol HClO/mL in the control group and 320.69 ± 53.634 µmol HClO/mL in the treatment group (Figure 2a). Consequently, the dROMs test shows no significant differences at day 42 in the CTRL and TRT groups (145.44 ± 36.511 and 182.85 ± 19.297 UCARR, respectively) (Figure 2b).

#### 3.2.3. Immunoenzymatic Test

As well as the previous parameters considered, the enzyme immunoassays performed showed no significant differences between the two test groups. Specifically, DAO decreased during the trial in both experimental groups, and TFF-3 did not differ after 42 days between the CRTL and TRT group (Table 4).

### 3.3. Diarrhea Occurrence and Fecal Samples Analysis

**Figure 3:**
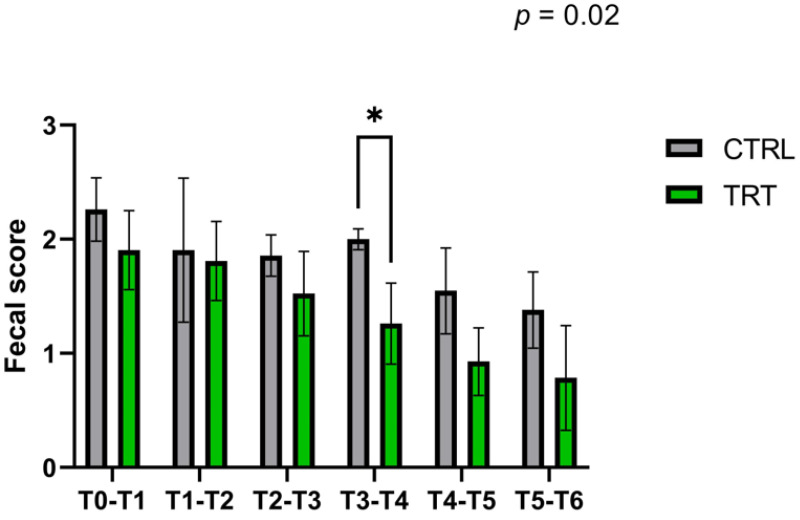
Average weekly fecal score in treatment (TRT) and control (CRTL) groups during 42 days of the experimental trial. T0–T1 = days 0–7, T1–T2 = days 7–14, T2–T3 = days 14–21, T3–T4 = days 21–28, T4–T5 = days 28–35, T5-T6 = days 35–42. * Asterisk indicates statistically significant differences between groups (*p* < 0.05). Data are presented as mean ± standard deviation.

The occurrence of diarrhea, assessed by fecal score determination, was found to have a constant trend throughout the trial period (Figure 3). Specifically, the data obtained showed that the TRT group had a reduced fecal score during all weeks of the trial compared with the CTRL group. This difference was also statistically significant (*p* < 0.05) during the fourth week of the trial.

**Figure 4:**
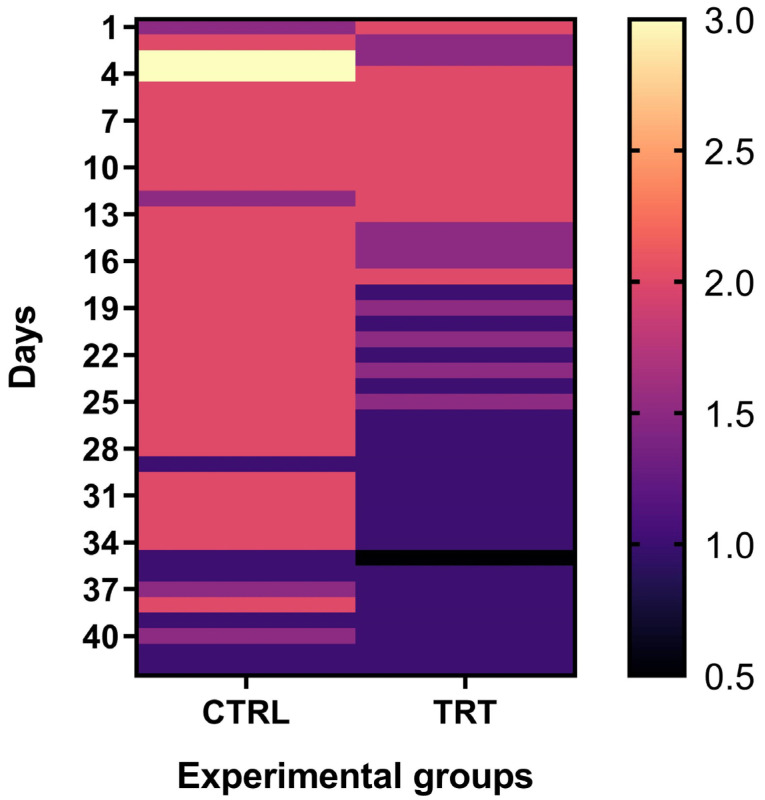
Daily distribution of fecal score in treatment and control groups, from day 0 to day 42. Darker colors indicate greater fecal consistency = minor cases of diarrhea; lighter colors indicate minor fecal consistency = greater prevalence of diarrhea.

These findings in lower fecal score value in the TRT group align with the registered days of diarrhea occurrence. Figure 4 shows the daily distribution of fecal scores between the two groups. Darker colors indicate a low fecal score with a lower incidence of diarrhea. In comparison, lighter colors are associated with higher fecal scores and a higher incidence of diarrhea and the critical period (fecal score over 2) in the CRTL group lasted over 34 days (*p* < 0.024). In contrast, in the TRT group, the fecal score began to drop at around day 19, with a more excellent distribution of more compact feces.

**Figure 5:**
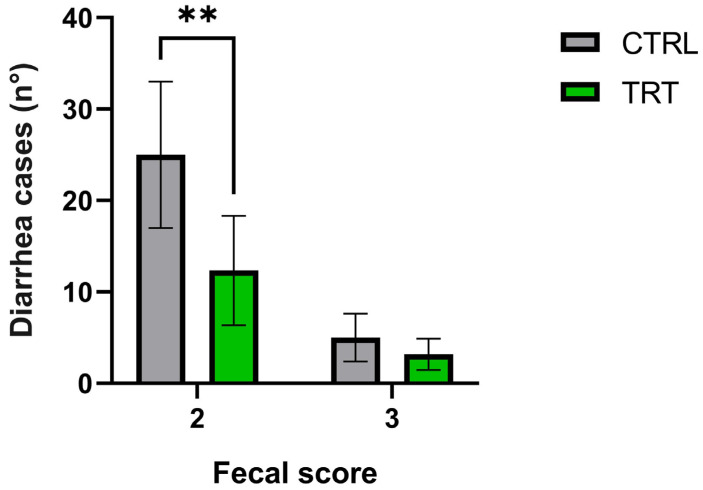
Total diarrhea cases recorded during the 42-day trial for the control (CTRL) and treatment groups (TRT). Data are expressed as the sum of recorded cases of diarrhea measured daily, considering: (a) Cases of moderate diarrhea, fecal score (FS) = 2; (b) Cases of severe diarrhea, fecal score (FS) = 3. ** Asterisks indicate statistically significant differences between groups (*p* = 0.0024). ** *p*-value < 0.05 for fecal score 2 (moderate diarrhea) and no differences for fecal score 3 (severe diarrhea).

The fecal scores were further evaluated by adding the total number of cases of moderate diarrhea (fecal score 2) and severe diarrhea (fecal score 3) throughout the trial (Figure 5). There was a difference between the two groups in cases of fecal score 2, where the animals showed a reduction of moderate diarrhea of 50.66% in TRT compared to CTRL during the experimental trial, but there were no significant differences in severe diarrhea cases (FS 3). The number of total days with fecal score = 2, considering all the animals, was 150 days for the CRTL group and 74 days for the TRT group, and were significantly lower in the TRT group, suggesting a possible ability of *A. nodosum* to counteract the onset of moderate diarrhea (Figure 5) [33,34].

### 3.4. Bacterial Count in Fecal Samples

**Figure 6:**
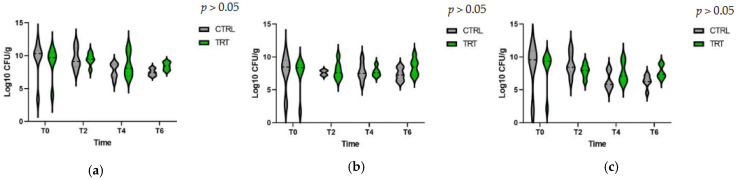
Bacteria count, total bacteria, lactic acid, and coliform bacteria at T0 = day 0, T2 = day 14, T4 = day 28, and T6 = day 42. CRTL: control group, TRT: treatment group. (**a**) total bacteria count, (**b**) lactic acid bacteria count, and (**c**) coliform bacteria count.

No differences between groups have been revealed regarding total bacteria, lactic acid, and coliform bacteria count; high individual variability is observed during the trial (Figure 6).

### 3.5. PCR, Molecular Microbiology

**Figure 7:**
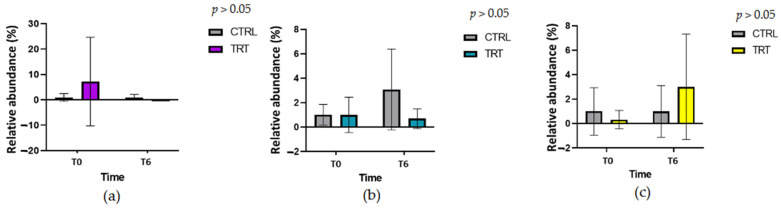
Target DNA of principal microbial indicators of gut microbiota in control and treatment groups at day 0 (T0) and day 42 (T6). (**a**) *Bifidobacterium* spp., (**b**) *E. coli*, (**c**) *Lactobacillus* spp.

The relative abundance of health-promoting and Gram-negative pathogenic bacteria in the feces was determined by real-time PCR. The values were normalized considering the CTRL group to have a value of 1. Figure 7 shows no statistical differences between *Bifidobacterium* spp., *Lactobacillus* spp., and *E. Coli* between CTRL and TRT groups (*p* > 0.05). 

## 4. Discussion

### 4.1. Zootechnical Performances

Zootechnical performances of calves are related to several factors, and can be implemented through correct management, that can involve, for example, new technological systems correlated to the innovative approach of precision livestock that aims to look at physiological, behavioral, and production indicators to improve management and performance [35], or nutritional aspects directly correlated with animal growth and future milk production [36]. Our results, related to zootechnical performance, showed no differences between the experimental groups. This result highlights that the inclusion of 10 g/day of *A. nodosum* did not affect the growth of animals. This is in line with previous studies reported in literature. In fact, studies reported that use a higher inclusion level (25 g/4L) did not disclose significant differences either [37]. Therefore, choosing a lower dosage of inclusion is a good strategy from a cost/benefit ratio perspective. Average daily feed intakes (ADFI), along with body weight (BW), and the feed conversion ratio (FCR), are commonly used for evaluating the zootechnical performances of animals during feeding trials. An animal’s growth can be considered an indirect index of its health status, where a phenotypical evaluation correlates to animal welfare. Our results on the performances of calves that were fed with *A. nodosum* align with other studies [37], where the zootechnical performance did not differ between the two groups. BW and ADFI constantly increased during the trial, in line with previous studies in each group [38].

### 4.2. Metabolic Profile, Antioxidant Barrier, and Immunoenzymatic Analysis of Serum Samples

Metabolic profile, antioxidant barrier, and immunoenzymatic evaluations in calves provided additional data on their health status. Metabolic profile differed between groups. Albumin is produced in the liver, and is a serum protein involved in osmotic pressure maintenance. Its principal role is the metabolite transport of, for example, calcium, lipids, hormones, and drugs. The increase in albumin could be directly correlated with the presence of calcium, phosphorus, and total cholesterol because albumin is directly related to their transport. At different days of life, a significant modification in total protein, albumin, and globulin levels has been observed (*p* < 0.001) [39]. In previous work [39], albumin constantly increased significantly from birth up to day 84 of age (*p* < 0.001). This indicator partially reflects hepatic synthesis and could be related to compensation of decreasing serum osmotic pressure due to a decline in globulin levels. In our study, after 42 days (*p* < 0.05), the TRT group exhibited an increased concentration of total cholesterol and no difference in triglyceride levels compared with the CTRL group. The quantity of fat absorbed from the gastrointestinal tract (GIT) and re-esterified lipids (in various lipoproteins) into low-density lipoproteins (LDL) by the liver determines the cholesterol serum levels in calves [40]. *A. nodosum* increased lipid metabolism in rats, thereby promoting antioxidant activity [41]. One of the principal mineral elements in *A. nodosum* is iodine, which is related to higher levels of total cholesterol. This mineral in animal nutrition is involved in the activation of energy metabolism [42]. The hypometabolic profile represented by the whole cholesterol level was thus different among groups [26]; the treated group had a greater total cholesterol concentration than the control group. The higher levels in the treated group could result from higher systemic metabolic activity due to the mineral and functional characteristics of *A. nodosum* [43]. There were different minerals in the two study groups due to the high mineral content of *A. nodosum*, especially iodine, calcium, phosphorus, and potassium [43,44]. An increase in circulating calcium and phosphorus levels in the bloodstream of the treated group could be related to the supplementation with *A. nodosum*, which provides an increased level of micronutrients. In addition, calcium and phosphorus homeostasis reveals the animal’s healthy bone structures, which were under the hormonal control of calcitonin and parathormone [45]. High circulating levels suggested ample mineral availability and, thus, an increased basal metabolism in favor of free calcium and phosphorus. In particular, albumin is directly correlated with diarrhea presence. Previous studies disclosed that the animals with diarrhea had lower albumin values compared to those without diarrhea, thus supporting the correlation between the albumin value and the diarrhea cases [46]. Calves with diarrhea often have decreased levels of total cholesterol. These changes in lipid levels are thought to be due to the liver’s increased cholesterol metabolism in response to diarrhea. The liver produces bile acids from cholesterol, which are then used to digest fats in the small intestine. When calves have diarrhea, they lose fluids and electrolytes, including bile acids. This can lead to decreased cholesterol levels in the blood [47]. The higher level of calcium and phosphorus observed in the TRT group could be correlated to the inclusion of algae in the milk replacement. *A. nodosum* is rich in minerals and reports a high level of calcium and phosphorus [44]. Regarding oxidative status, the obtained outcomes in the oxidative group of blood serums suggest that the algae supplementation did not impair the serum antioxidant barrier, thus highlighting the overall good health status of the animals. Regarding the immunoenzymatic test, DAO and TFF-3 were chosen as indirect markers of the gastrointestinal integrity of the calves, and no differences were observed in either of the results. The treatment did not influence these parameters. The TFF-3 was considered in this study as a marker of the calves’ diarrhea status, as it has been shown that a higher serum concentration of this biomarker is correlated with diarrhea in calves [48]. The increase in TFF-3 in serum is also associated with mucosal damage [45]. DAO is an enzyme that has demonstrated, like TFF-3, a correlation with intestinal wall permeability and, consequently, the barrier’s functionality [45]. DAO is implicated in the degradation in the small intestine of histamine, a cytoplasmatic enzyme localized in the mucosa. This enzyme in serum helps assess diarrhea in humans, rats, and calves [49].

### 4.3. Diarrhea Occurrence and Fecal Samples Analysis

Fecal score (FS) 0–1 was considered as an index of normal feces, while FS 2–3 showed a watery consistency of waste (considered diarrhea) [25,50,51]. Different management, hygiene, and colostrum assumptions can modify the duration of passive immunity [52]; after the initial period of life, the animals gradually reduce passive immunity [53]. The previous finding could be due to the supplementation of *A. nodosum*, which has shown an interesting antimicrobial effect against several pathogens involved in diarrhea [13]. In addition, the period between T3–T4 corresponds to the acquirement of active immunity by calves, which in TRT probably resulted in more resilient animals with improved fecal consistency. *A. nodosum* is a functional ingredient that reduces mild diarrhea cases, not acting on severe diarrhea. The results suggested a preventive effect of algal supplementation in mild diarrhea cases (Figure 5).

### 4.4. Bacterial Count in Fecal Samples

Immediately after calving, the newborn colonizes their gastrointestinal tract, the starting point of developing microbiota, until the gut microbiota stabilizes with animal growth [54]. Weaning calves are particularly subject to gastrointestinal infections and diarrhea is often associated with pathogens. Nevertheless, an alteration in the gut microbial population plays an important role. As a consequence, we assessed the principal fecal microbial classes.

### 4.5. PCR, Molecular Microbiology

Our plate counting method resulted in an overall indication of the principal bacterial composition in feces. We also conducted a molecular approach based on qPCR for fecal samples to identify the principal bacterial population. In our study on calves, the counting method and q-PCR disclosed similar results. A slight increase in *Lactobacillus* spp. in the TRT group at T6 has been observed. On the other hand, a slight increase in *E. coli* was observed in the CTRL group simultaneously. This suggests that adding *A. nodosum* to the milk replacer does not impair the fecal bacterial composition in calves. However, more studies are needed to increase the sample size population that, in the present study, could be a limitation due to the smallness of the sample size. Moreover, these data should be considered preliminary. In fact, for a more profound knowledge of the effects of *A. nodosum* supplementation on the intestinal microbiota composition, it is necessary to define the interaction in the gut microbiota. Previous studies on tributyrin in piglets showed a reduction in the *Lactobacillus* spp. [55], and a significant increase in the beta diversity of gut microbiota with a positive modulation of several bacteria, which are generally positively correlated with animal performance and health.

## 5. Conclusions

The dietary supplementation of *A. nodosum* in a milk replacer of Holstein Frisian calves during the pre-weaning period revealed an improvement in the fecal score in the treated group, thus suggesting a possible counteracting effect, particularly in terms of reducing the moderate diarrhea frequency. Differences in the serum metabolic profile were found in the concentrations of albumin, total cholesterol, calcium, and phosphorus, which were higher in the treated group, suggesting a positive effect on animal health, such as the correlations of albumin and cholesterol with diarrhea. Therefore, our study highlights that supplementing *A. nodosum* in pre-weaning calves could be used as a method in disease prevention strategies and in improving health status according to the One Health principles.

## Figures and Tables

**Table 1:** Nutritional composition of milk replacer (data provided by the producer: Gruppo Veronesi S.p.a., Italy).

Analyte	Composition (% as Fed)
Crude protein	23.00
Ether extract	18.00
Crude fiber	0.10
Ash	7.50
Lys	2.10
Ca	1.00
P	0.70
Na	0.50

Additives per kg: vitamins, pro-vitamins, and substances with similar effects: vitamin A 20,000 IU; vitamin D3 4000 IU; vitamin E 100 mg; vitamin C 150 mg; vitamin B1 6 mg; vitamin B2 12 mg; vitamin B6 6 mg; vitamin B12 80 mg; niacin 30 mg; calcium D-pantothenate 25 mg; vitamin K3 4 mg; betaine hydrochloride 250 mg; trace elements: iron 75 mg; copper 6 mg; zinc 85 mg; iodine 1 mg; manganese 30 mg; selenium 0.3 mg.

**Table 2:** Sequence of nucleotide used for the bacteria strain detection by PCR.

Target	Forward/Reverse	Nucleotide Sequence	Amplicon (bp)	Reference
*Lactobacillus* spp.	Fw	5′–CTTGTACACACCGCCCGTCA–3′	250	[29]
	Rv	5′–CTCAAAACTAAACAAAGTTTC–3′		
*Bifidobacterium* spp.	Fw	5′–CTCCTGGAAACGGGTGG–3′	549–563	[30]
	Rv	5′–GGTGTTCTTCCCGATATCTACA–3′		
*E. coli*	Fw	5′–ATGCTTAGTGCTGGTTTAGGG–3′	248	[31]
	Rv	5′–GCCTTCATCATTTCGCTTTC–3′		
Total bacteria	Fw	5′–CGGCAACGAGCGCAACCC–3′	130	[32]
	Rv	5′–CCATTGTAGCACGTGTGTAGCC–3′		

**Table 3:** Metabolic profile of serum samples in treatment (TRT) and control (CRTL) groups after 42 days of the trial.

Analyte	LSMeans		SE	*p*-Value
	CTRL	TRT		
Albumin (g/L)	41.96 *	48.43 *	1.374	0.014 *
Albumin/globulin (A/G)	1.22	1.25	0.090	0.869
Beta-hydroxyb-utyrate (mmol/L)	0.07	0.09	0.010	0.165
Calcium (mmol/L)	3.75 *	4.48 *	0.161	0.018 *
Gamma-glutamyl transferase (IU/L)	39.42	45.75	5.212	0.414
Globulin (g/L)	35.62	39.73	3.079	0.436
Glucose (mmol/L)	8.36	9.66	0.491	0.118
Magnesium (mmol/L)	1.17	1.26	0.052	0.296
Non-esterified fatty acid (mmol/L)	0.67	0.66	0.073	0.207
Phosphorus (mmol/L)	4.01 *	4.84 *	0.170	0.012 *
Total bilirubin (µmol/L)	5.56	6.44	4.770	0.267
Total cholesterol (mmol/L)	4.47 *	6.61 *	0.392	0.006 *
Total protein (g/L)	77.51	88.27	4.019	0.133
Triglycerides (mmol/L)	0.81	0.98	0.100	0.260
Urea (mmol/L)	2.72	2.78	0.233	0.842

* Asterisk indicates statistically significant differences (*p*-value < 0.05).

**Table 4:** Serum concentration of diamine oxidase (DAO) and trefoil factor 3 (TFF-3) in control (CTRL) and treatment (TRT) groups at 0 and 42 days of the trial.

Mean ± SD	CTRL T6	TRT T6
DAO	21.11 ± 1.972	18.56 ± 3.504
TFF-3	1.61 ± 0.523	1.70 ± 0.202

Results are presented as mean and standard deviation. T6 = day 42. *p* > 0.05.

## Data Availability

The data presented in this study are not deposited in an official repository. Data are available within the article and from the corresponding author upon reasonable request.
